# The effect of metabolic dysfunction-associated fatty liver disease and diabetic kidney disease on the risk of hospitalization of heart failure in type 2 diabetes: a retrospective cohort study

**DOI:** 10.1186/s13098-023-01006-z

**Published:** 2023-03-01

**Authors:** Seung Eun Lee, Juhwan Yoo, Bong-Seong Kim, Han Seok Choi, Kyungdo Han, Kyoung-Ah Kim

**Affiliations:** 1 Division of Endocrinology and Metabolism, Department of Internal Medicine, Dongguk University Ilsan Hospital, 27, Dongguk-ro, Ilsandong-gu, Goyang-si, Gyeonggi-do South Korea; 2 Department of Biomedicine & Health Science, The Catholic University of Korea, Seoul, South Korea; 3 Department of Statistics and Actuarial Science, Soongsil University, 369, Sangdo-ro, Dongjak-gu, Seoul, South Korea

**Keywords:** Heart failure, Diabetic kidney disease, Metabolic dysfunction–associated fatty liver disease, Hospitalization for heart failure

## Abstract

**Background:**

Diabetes mellitus is a major risk factor for heart failure. A recent consensus statement recommended annual cardiac biomarker testing (e.g. natriuretic peptide or high-sensitivity cardiac troponin) for all patients with diabetes. We aimed to identify patients at a higher risk of hospitalization for heart failure among patients with type 2 diabetes to prioritize those who would require screening.

**Methods:**

Overall, 1,189,113 patients who underwent two medical health checkup cycles (2009–2012 and 2011–2014) and had stable diabetic kidney disease (DKD) phenotype in the Korean National Health Insurance Service database were included in this study. After excluding those with concurrent proteinuria (PU) and reduced estimated glomerular filtration rate, three groups (no-DKD, PU^+^DKD, and PU^−^DKD) were identified. A fatty liver index of ≥ 60 was defined as metabolic dysfunction–associated fatty liver disease (MAFLD). Patients were followed up until December 2018 or until outcomes developed. The Cox proportional hazard model was used to compare the risk of hospitalization for heart failure across groups.

**Results:**

During an average of 6.6 years of follow-up, 5781 patients developed hospitalization for heart failure. After adjusting for covariates, the risk of hospitalization for heart failure was highest in the PU^+^DKD group [HR 3.12, 95% CI (2.75–3.55)], followed by the PU^−^DKD group [HR 1.85, 95% CI (1.73–1.99)] using the no-DKD group as the reference category. The risk of hospitalization for heart failure was comparable regardless of MAFLD status in patients who already had DKD. However, in the no-DKD group, the risk of hospitalization for heart failure was 1.4 times higher in patients with MAFLD than in those without [HR 1.41, 95% CI (1.31–1.52)].

**Conclusions:**

In lines with the international consensus statement, we suggest that annual cardiac biomarker testing should be conducted at least in patients with DKD and/or MAFLD.

**Supplementary Information:**

The online version contains supplementary material available at 10.1186/s13098-023-01006-z.

## Background

Heart failure (HF) is a global health problem with a rising prevalence rate [1, 2] mostly due to prolonged human lifespan. Worldwide, HF affects approximately 26 million people [3] and the burden is estimated to increase continuously. Mortality and morbidity associated with HF are high, and the mortality rate of HF was reported to be approximately 10% at 30 days, 20–30% at 1 year, and 45–60% over 5 years of follow-up [4]. HF is attributed to cumulative exposure to multiple risk factors; hence, early screening, detection, and correct modifiable risk factors are essential for reducing HF-related burden [2].

Type 2 diabetes is a well-known risk factor for HF [5]. Based on a report that shows many people with diabetes have subclinical structural heart disease, the American Diabetes Association (ADA) recommends measuring natriuretic peptide or high-sensitivity cardiac troponin in patients with diabetes on at least a yearly basis [6]. However, it may be impractical to perform biochemical tests for all patients with diabetes, and risk stratification with specific clinical recommendations should be provided. Chronic kidney disease (CKD) is a well-established risk factor for HF [7]. A growing body of evidence has recently shown the association between nonalcoholic fatty liver disease (NAFLD) and HF [8, 9]. In 2020, a new nomenclature for metabolic dysfunction–associated fatty liver disease (MAFLD) was proposed as a substitute for NAFLD [10], and this definition was endorsed by global multi-stakeholder [11]. It may be useful for identifying a greater number of individuals with metabolically complicated fatty liver and an increased risk for cardiovascular disease (CVD) [12, 13].

Several multivariable models have been used to predict the risk of HF in patients with diabetes [6, 14]. Although renal dysfunction was included as a risk factor for HF in reported models, none of them considered MAFLD. Therefore, in this study, we investigated whether the inclusion of diabetic kidney disease (DKD) and/or MAFLD improves the prediction of HF risk in patients with type 2 diabetes.

## Methods

### Data source and study population

This study used data from the Korean National Health Insurance Service (NHIS), which is the sole insurance provider for all Korean residents. The NHIS-established databases (DBs) included the qualification, treatment, and medical checkup DBs [15]. Briefly, the qualification DB included data on qualifications, including age, sex, location, and socioeconomic variables; the treatment DB contained payment data to the clinic upon treatment of the patients at the clinic; and the medical checkup DB comprised major results from medical checkups, behavior, and habitual data from the questionnaire. We used the qualification and medical checkup DBs to examine the baseline characteristics of the study population and the treatment DB to investigate the outcomes. Because we used previously collected, publicly available, de-identified data, ethical review by the Institutional Review Board and informed consent were exempted. Permission for the use of health check-up data was granted by the NHIS (NHIS-2021-1-634).

### Study design

This study included 1,779,819 subjects with type 2 diabetes who underwent at least two general medical checkups in 2009–2012 and 2011–2014 (Additional file 1: Fig. S1A). The exclusion criteria were as follows: (1) individuals diagnosed with cancer (n = 68,282); (2) individuals diagnosed with thyrotoxicosis (n = 78,467); (3) individuals with renal diseases other than DKD (n = 135,698); (4) individuals with rheumatic mitral valve disease (n = 4695); (5) individuals with missing values (n = 48,959); and (6) those with an estimated glomerular filtration rate (eGFR) of less than 30 mL/min/1.73 m^2^ (n = 14,889). Additionally, patients with proteinuric DKD with reduced eGFR (< 60 mL/min/1.73 m^2^) at the first examination were excluded since the very high-risk Kidney Disease: Improving Global Outcomes (KDIGO) categories are well known for poor cardiovascular outcomes [16]. Subsequently, we only included patients with a stable DKD phenotype for over 2 years.

Patients with a stable DKD phenotype for over 2 years were subclassified according to the presence or absence of MAFLD and were followed up until hospitalization for heart failure (HHF) or December 2018 (Additional file 1: Fig. S1B).

### Definitions of diabetes, DKD, and MAFLD

Type 2 diabetes was defined as the presence of the diagnostic code (International Classification of Disease-Tenth Revision (ICD-10) code: E11–E14) and the prescription of relevant glucose-lowering drugs. When the participants did not meet the criteria above, they were defined as having type 2 diabetes if their fasting plasma glucose levels were ≥ 126 mg/dL during a medical checkup.

The eGFR was determined using the equation from the Modification of Diet in Renal Disease study [17] and reduced eGFR was defined as values less than 60 mL/min/1.73 m^2^. Notably, positive proteinuria (PU) of ≥ 1 + was defined based on the urinary dipstick test. Additionally, the DKD phenotype was categorized into three distinct groups based on the eGFR levels (normal vs. reduced) and PU (negative vs. positive) as follows: group 1 (no-DKD), normal eGFR and negative PU; group 2 (PU^+^DKD), normal eGFR and positive PU; and group 3 (PU^−^DKD), reduced eGFR and negative PU.

The fatty liver index (FLI) was used to identify patients with MAFLD [18]. According to the criteria [18], MAFLD was diagnosed regardless of having other etiologies such as alcohol-associated fatty liver disease and viral hepatitis. FLI was calculated using the following equation: e^x^/(1 + e^x^) × 100, x = 0.953 × log (triglyceride) + 0.139 × body mass index + 0.718 × log (gamma-glutamyl transferase (GGT)) + 0.053 × waist circumference − 15.745). Particularly, an FLI of ≥ 60 was defined as MAFLD [19].

### Definitions of comorbidities

Patients with HF were identified based on ICD-10 codes for heart failure (I50). Hypertension was indicated in patients according to the ICD-10 code for hypertension (I10–I13, I15) and the prescribed antihypertensive medications. Participants were also considered hypertensive if their systolic blood pressure was ≥ 140 mmHg and/or diastolic blood pressure was ≥ 90 mmHg during a general medical checkup. Moreover, patients with dyslipidemia were identified by the ICD-10 code for dyslipidemia (E78) with treatment undergone using lipid-lowering agents or a total cholesterol level ≥ 240 mg/dL during a medical checkup. Proliferative diabetic retinopathy (PDR) was established if the participants had two or more diagnoses of diabetic retinopathy (H360) and a procedure code for pan-retinal photocoagulation (S5160, S5161).

### Laboratory and clinical examination

This study obtained laboratory results and clinical characteristics during the second examination. Body mass index was calculated as weight divided by height in meters squared (kg/m^2^). After overnight fasting, venous samples were used to evaluate fasting plasma glucose, total cholesterol, triglyceride, high-density lipoprotein cholesterol, low-density lipoprotein cholesterol, creatinine, aspartate aminotransferase (AST), alanine aminotransferase (ALT), GGT, and hemoglobin levels.

Additionally, health-related lifestyles were evaluated using self-administered questionnaires categorized as current smokers or non-smokers, heavy drinkers (≥ 30 g/day of alcohol) or non-heavy drinkers, and participants with or without regular exercise.

### Outcome

The primary outcome of this study was the hospitalization of patients for HF. Cases were defined as patients who were admitted to a hospital with a discharge diagnostic code for HF (I50).

### Statistical analysis

Descriptive statistics were used to summarize baseline characteristics. Baseline characteristics across the groups were presented as numbers (percentages) for categorical variables and mean ± standard deviation for continuous variables. If the distribution of continuous variables was heavily skewed, the geometric mean was used. To analyze the differences in baseline characteristics between the groups, a one-way analysis of variance was used for continuous variables, and the chi-squared test was used for categorical variables.

The cumulative incidence of HHF was calculated using Kaplan–Meier estimates, and we performed a log-rank test to analyze the differences in HHF risk across groups. The incidence of HHF was expressed as the number of events per 1000 person-years. Cox proportional hazards regression analysis was performed to assess the hazard ratio (HR) for HHF across the groups. Model 1 was unadjusted; Model 2 was adjusted for age and sex; and Model 3 was adjusted for smoking, drinking, and physical activity. Additionally, Model 4 was adjusted for comorbidities, including hypertension, dyslipidemia, atrial fibrillation, and ischemic heart disease. Finally, Model 5 was further adjusted for fasting glucose, diabetes duration, hemoglobin levels, and insulin usage. Subgroup analyses with tests for interaction were performed according to age group (< 65 vs. ≥ 65 years), sex, and the presence or absence of prevalent HF. Statistical analyses were conducted using the SAS software (version 9.4; SAS Institute, Cary, NC, USA). Statistical significance was set at *P* < 0.05.

## Results

### Baseline characteristics of the study population

Table 1 presents the baseline characteristics of the study population according to changes in the DKD phenotype. The prevalence of DKD phenotypes was 95.2% (1,132,531/1,189,113) in the no-DKD group, 1.3% (15,619/1,189,113) in the PU^+^DKD group, and 3.4% (40,963/1,189,113) in the PU^−^DKD group. The prevalence of MAFLD was 25.1% (298,522 of 1,189,113), and patients with MAFLD were younger, more obese, and more likely to be male than those without MAFLD. Additionally, they tended to be current smokers and heavy drinkers and did not exercise regularly. The prevalence of hypertension and dyslipidemia was higher in patients with MAFLD than in those without MAFLD. Despite the shorter duration of diabetes, patients with MAFLD showed higher fasting plasma glucose levels than those without MAFLD. Predictably, the AST, ALT, and GGT levels were higher in patients with MAFLD than in those without MAFLD.

**Table 1:** Baseline characteristics according to DKD/MAFLD phenotype

	no-DKD (n = 1,132,531)		PU^+^DKD (n = 15,619)		PU^−^DKD (n = 40,963)		
	MAFLD−	MAFLD+	MAFLD−	MAFLD+	MAFLD−	MAFLD+	
n	848,716	283,815	9107	6512	32,768	8195	
Male	507,170 (59.76)	231,817 (81.68)	6670 (73.24)	5390 (82.77)	12,627 (38.53)	4475 (54.61)	< 0.001
Age (years)	57.48 ± 11.88	52.62 ± 11.3	60.14 ± 10.45	53.79 ± 10.75	70.62 ± 8.09	67.96 ± 8.79	< 0.001
BMI (mg/k^2^)	23.89 ± 2.62	27.91 ± 3.23	24 ± 2.61	28.3 ± 3.62	24.27 ± 2.69	28.56 ± 3.19	< 0.001
WC (cm)	82.17 ± 7.09	92.86 ± 7.38	83.72 ± 6.81	94.17 ± 8.05	83.98 ± 7.28	95.46 ± 7.22	< 0.001
Current smoker	198,817 (23.43)	109,561 (38.6)	2613 (28.69)	2558 (39.28)	2883 (8.8)	1137 (13.87)	< 0.001
Heavy drinker	58,984 (6.95)	54,637 (19.25)	805 (8.84)	1380 (21.19)	599 (1.83)	517 (6.31)	< 0.001
Regular Exercise	200,956 (23.68)	55,512 (19.56)	2138 (23.48)	1259 (19.33)	6648 (20.29)	1508 (18.4)	< 0.001
Comorbidities							
Hypertension	409,799 (48.28)	169,157 (59.6)	6818 (74.87)	5118 (78.59)	26,963 (82.28)	7234 (88.27)	< 0.001
Dyslipidemia	322,792 (38.03)	130,242 (45.89)	4819 (52.92)	3891 (59.75)	18,350 (56)	4939 (60.27)	< 0.001
IHD	126,334 (14.89)	37,077 (13.06)	1911 (20.98)	1144 (17.57)	10,708 (32.68)	2689 (32.81)	< 0.001
AF	7394 (0.87)	2295 (0.81)	171 (1.88)	106 (1.63)	936 (2.86)	273 (3.33)	< 0.001
Stroke	48,612 (5.73)	11,547 (4.07)	923 (10.14)	394 (6.05)	5466 (16.68)	1199 (14.63)	< 0.001
PAD	147,245 (17.35)	39,420 (13.89)	2151 (23.62)	1111 (17.06)	10,198 (31.12)	2422 (29.55)	< 0.001
CVD	256,905 (30.27)	71,782 (25.29)	3815 (41.89)	2111 (32.42)	18,938 (57.79)	4561 (55.66)	< 0.001
Heart failure	18,840 (2.22)	5978 (2.11)	357 (3.92)	201 (3.09)	2917 (8.9)	772 (9.42)	< 0.001
Severity of diabetes							
FPG ≥ 150 mg/dL	175,770 (20.71)	84,175 (29.66)	3787 (41.58)	3340 (51.29)	6040 (18.43)	2022 (24.67)	< 0.001
FPG (mg/dL)	131.11 ± 41.77	141.98 ± 45.4	154.7 ± 58	164.19 ± 53.07	127.41 ± 43.07	136.17 ± 46.43	< 0.001
DM ≥ 5 yrs	297,221 (35.02)	65,008 (22.91)	5938 (65.2)	2803 (43.04)	20,628 (62.95)	4365 (53.26)	< 0.001
Insulin use	59,635 (7.03)	13,919 (4.9)	1997 (21.93)	835 (12.82)	5445 (16.62)	1256 (15.33)	< 0.001
≥ 2 oral GLD	346,564 (40.83)	102,721 (36.19)	5862 (64.37)	3615 (55.51)	18,700 (57.07)	4541 (55.41)	< 0.001
PDR	3751 (0.44)	437 (0.15)	346 (3.8)	99 (1.52)	359 (1.1)	50 (0.61)	< 0.001
SBP (mmHg)	126.3 ± 14.78	131.15 ± 14.51	132.64 ± 16.82	136.69 ± 16.86	129.64 ± 16.05	132.05 ± 16.01	< 0.001
DBP (mmHg)	77.37 ± 9.59	81.7 ± 9.87	79.46 ± 10.53	84.18 ± 11.1	76.38 ± 10.17	79 ± 10.17	< 0.001
eGFR (mL/min/1.73m^2^)	91.69 ± 36.74	92.26 ± 40.4	87.31 ± 35.03	91.7 ± 46.9	50.58 ± 7.29	50.78 ± 7.28	< 0.001
Non HDL-C (mg/dL)	136.22 ± 38.54	155.02 ± 43.5	139.24 ± 43.11	160.8 ± 51.24	132.94 ± 40.33	147.64 ± 57.7	< 0.001
AST (IU/L)*	23.75 (23.73–23.77)	32.63 (32.57–32.69)	23.57 (23.38–23.76)	33.44 (33.01–33.87)	23.14 (23.06–23.23)	28.61 (28.32–28.9)	< 0.001
ALT (IU/L)*	22.6 (22.58–22.63)	37.86 (37.78–37.94)	22.52 (22.29–22.75)	36.85 (36.33–37.38)	19.03 (18.94–19.13)	27.6 (27.26–27.94)	< 0.001
γGTP (IU/L)*	27.78 (27.74–27.81)	74.51 (74.3–74.72)	31.27 (30.86–31.69)	78.87 (77.37–80.39)	22.79 (22.65–22.92)	49.13 (48.33–49.94)	< 0.001

no-DKD: normal eGFR (eGFR ≥ 60) with negative PU; PU^+^DKD: normal eGFR with positive PU; PU^−^DKD: reduced eGFR (eGFR < 60) with negative PU

Values are presented as mean ± standard deviation or number (%). Data for the parameters marked with an asterisk (*) are presented as the geometric mean and 95% confidence interval

AF: atrial fibrillation; ALT: alanine aminotransferase; AST: aspartate aminotransferase; BMI: body mass index; CVD: cardiovascular disease; DBP: diastolic blood pressure; DKD: diabetic kidney disease; DM: diabetes mellitus; eGFR: estimated glomerular filtration rate; FPG: fasting plasma glucose; γGTP: gamma-glutamyl transferase; GLD: glucose-lowering drugs; HDL-C: high-density lipoprotein cholesterol; IHD: ischemic heart disease; MAFLD: metabolic dysfunction–associated fatty liver disease; PAD: peripheral artery disease; PDR: proliferative diabetic retinopathy; PU: proteinuria; RAS: renin-angiotensin system; SBP: systolic blood pressure; WC: waist circumference

### Risk of HHF according to DKD phenotype

During a mean follow-up of 6.6 years, 5781 of 1,189,113 patients were hospitalized for HF. The incidence rate of HHF was highest in the PU^−^DKD group, followed by the PU^+^DKD and no-DKD groups (4.14, 2.64, and 0.60 per 1000 person-years among patients, respectively) (Additional file 1: Table S1). After age and sex adjustments, the risk of HHF was higher in the PU^+^DKD group than in the PU^−^DKD group (PU^+^DKD: HR = 4.25, 95% CI 3.75–4.82; PU^−^DKD: HR = 2.46, 95% CI 2.30–2.64 using a no-DKD group as the reference category). This difference remained consistent after adjusting for social factors (model 3) and comorbidities (model 4). Notably, after adjusting for factors associated with the severity of diabetes (model 5), the effect of DKD phenotypes on HHF persisted (PU^+^DKD: HR = 3.12, 95% CI 2.75–3.55; PU^−^DKD: HR = 1.85, 95% CI 1.73–1.99 using a no-DKD group as the reference category).

### Risk of HHF according to DKD/MAFLD phenotype

**Fig. 1:**
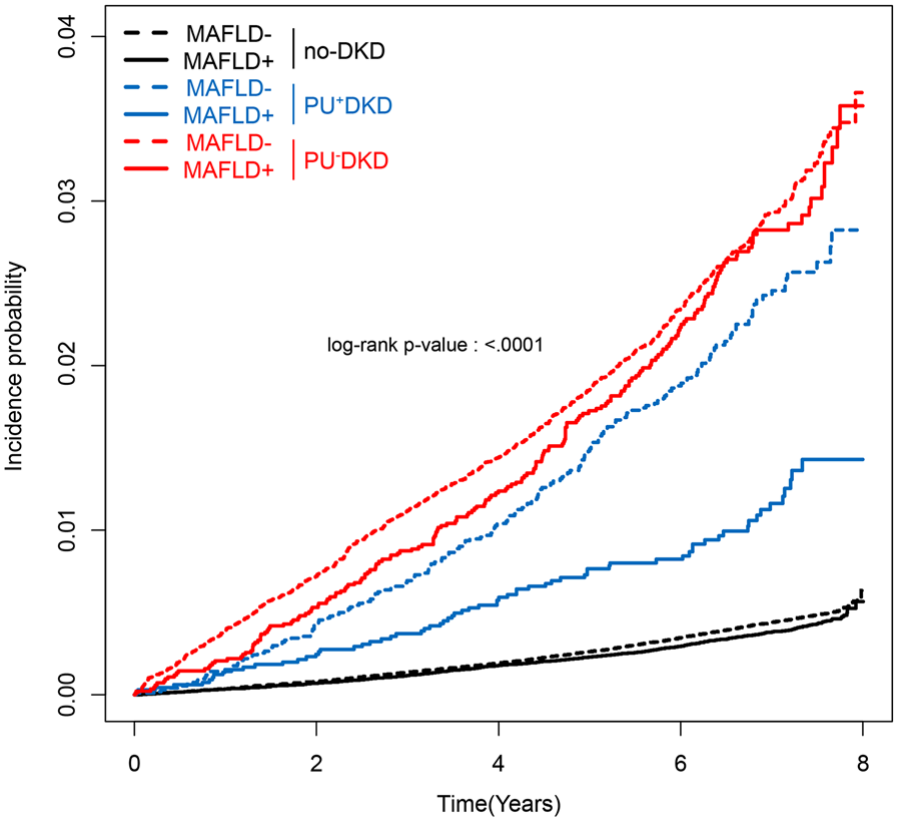
Cumulative incidence plot of hospitalization for heart failure according to the DKD/MAFLD phenotype. The black, blue, and red lines indicate the no-DKD, PU^+^DKD, and PU^−^DKD groups, respectively. The bold line indicates MAFLD and the dashed line indicates no MAFLD

**Fig. 2:**
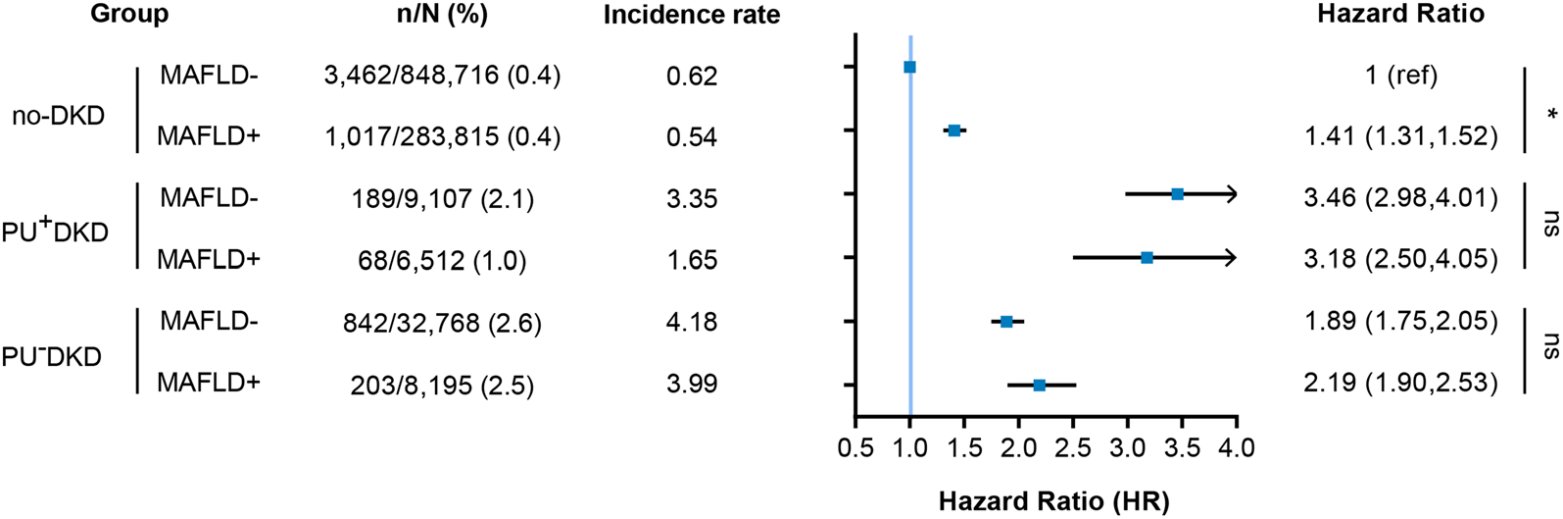
HRs and 95% CI of HHF according to DKD/MAFLD phenotype. DKD: diabetic kidney disease; HHF: hospitalization for heart failure; MAFLD: metabolic dysfunction–associated fatty liver disease; PU: proteinuria. no-DKD: normal eGFR (eGFR ≥ 60) with negative PU; PU^+^DKD: normal eGFR with positive PU; PU^−^DKD: reduced eGFR (eGFR < 60) with negative PU. HRs were adjusted for age, sex, smoking, alcohol, exercise, hypertension, dyslipidemia, atrial fibrillation, ischemic heart disease, fasting plasma glucose, diabetes duration, hemoglobin levels, and insulin usage

The cumulative incidence of HHF was higher in the MAFLD− group than in the MAFLD+ group (log-rank test, *P* < 0.001) (Fig. 1). However, after age and sex adjustment, the risk of HHF was comparable to or higher in patients in the MAFLD+ group than in those in the MAFLD- group (Additional file 1: Table S2). In the final models adjusting for potential confounding factors, the risk of HHF was significantly higher in no-DKD patients with MAFLD than in those without MAFLD (Fig. 2). Contrarily, the risk of HHF was comparable regardless of MAFLD status between the PU^+^DKD and PU^−^DKD groups (Fig. 2).

### Risk of HHF according to the FLI categories in the no-DKD group

**Fig. 3:**
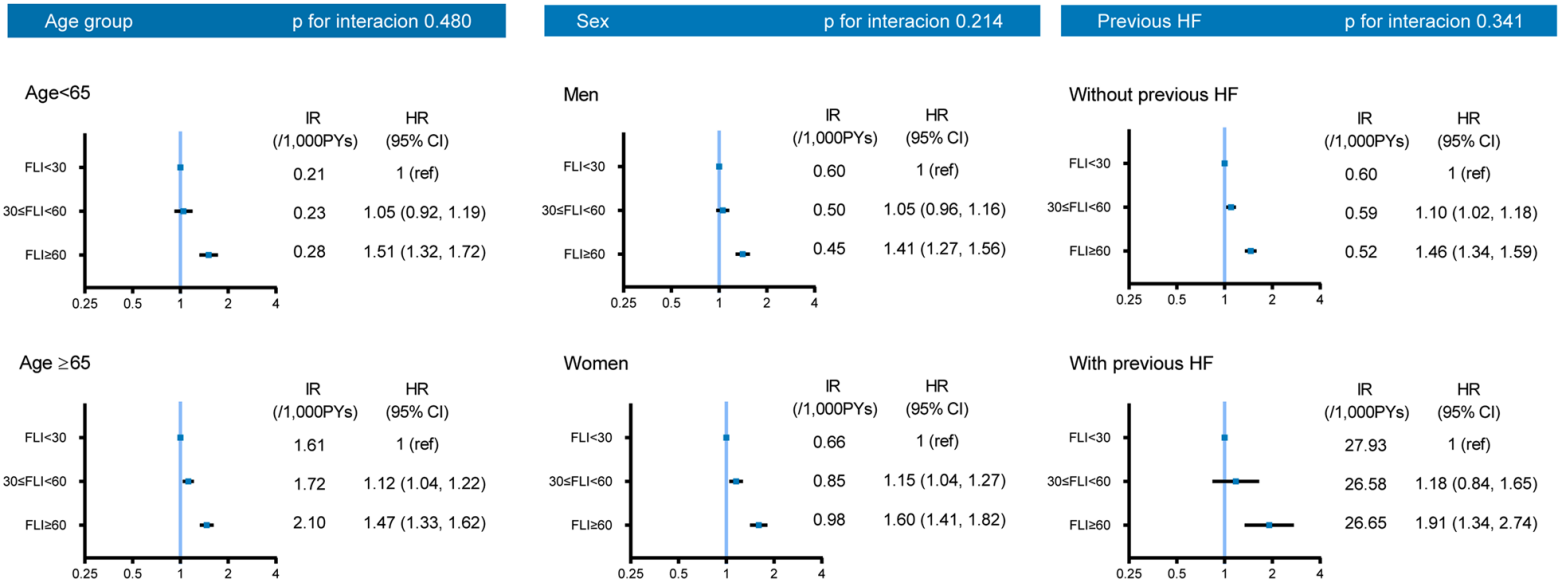
Subgroup analyses among no-DKD patients stratified by age, sex, and previous HF. DKD: diabetic kidney disease; FLI: fatty liver index; HR: hazard ratio; HF: heart failure; IR: incidence rate. HRs were adjusted for age, sex, smoking, alcohol, exercise, hypertension, dyslipidemia, atrial fibrillation, ischemic heart disease, fasting plasma glucose, diabetes duration, hemoglobin levels, and insulin usage

Previously, a lower FLI cutoff for diagnosing MAFLD has been suggested in the Korean population [20]. Consequently, we further analyzed the risk of HHF according to the three FLI categories (< 30 vs. 30–59 vs. ≥ 60). After adjustment for potential confounding factors, patients in the FLI > 60 and FLI 30–60 groups exhibited 1.5 times and 1.1 times, respectively, higher risk of HHF using an FLI < 30 group as reference (Additional file 1: Table S3). Additionally, subgroup analyses showed that the effect of FLI persisted regardless of age, sex, and the presence of previous HF (Fig. 3).

## Discussion

In this study, we observed that proteinuria and renal dysfunction were risk enhancers for HHF in patients with type 2 diabetes, which is consistent with previous studies [7]. In patients without proteinuria and reduced eGFR, MAFLD significantly increased the risk of HHF, suggesting that active diagnostic and interventional strategies should be provided for patients with diabetes, at least in those who concomitantly have DKD and/or MAFLD.

Although the 2022 ADA consensus report on HF has mandated annual cardiac biomarker testing for all patients with diabetes [6], the prevalence of HF and the healthcare system in each country might influence guideline approval. For instance, Korea has a relatively low prevalence of HF (1.53%) compared to Western countries (~ 2.2%) [21]. As a primary diagnosis, HF accounts for 0.78% of all hospital admissions in Korea compared to 3.04% in the USA [22]. Therefore, to identify patients at risk of HHF more precisely at the population level, we aimed to combine clinically available HHF risk enhancers, including fatty liver disease and CKD, to improve the implementation of guideline-directed medical therapy*.*

Notably, growing evidence suggests that individuals with MAFLD are at a higher risk of CKD [23] or cardiovascular disease than those with NAFLD [24]. The prevalence of MAFLD in patients diagnosed with the FLI was reported as 28.4% in a recent meta-analysis [25], and this is similar to the 25% prevalence of MAFLD observed in our study. Interestingly, the prevalence of MAFLD was the highest in PU^+^DKD (40%), followed by no-DKD (25%) and PU^−^DKD (20%). The highest MAFLD in PU^+^DKD is reminiscent of the severe insulin-resistant diabetes subtype, which is associated with an increased risk of fatty liver disease and macroalbuminuria [26].

Although the effects of NAFLD on CVD risk in patients with type 2 diabetes have been well established [27, 28], the association between NAFLD and incident HF has not been thoroughly explored. A meta-analysis showed that patients with NAFLD are 60% more likely to develop HF [29]. Similarly, we found increased HHF risk in patients with type 2 diabetes and MAFLD compared with those with type 2 diabetes without MAFLD; however, this was only observed in the absence of DKD. In the no-DKD group, a higher FLI score was significantly associated with a higher risk of HHF, which is consistent across subgroups (men or women; age ≥ 65 *or* < 65 years; previous HF yes or no).

CKD affects approximately 50% of patients with type 2 diabetes globally [30]. In addition, patients with type 2 diabetes and CKD are more likely to have diabetes-related complications, including cardiovascular morbidity [31]. Consistent with previous results, the risk of HHF events increased significantly in patients with type 2 diabetes and CKD compared with those with type 2 diabetes without CKD. Of note, the risk of HHF was higher in PU^+^DKD than in PU^−^DKD group. Normoalbuminuric DKD has become a widely prevalent variant of renal impairment in diabetes. Women, older, and nonsmoking individuals with good glycemic control have a better chance of preserving normoalbuminuria, even in the case of declining renal function [32]. Normoalbuminuric DKD, despite of a more favorable option in terms of the risk of end-stage renal disease, was reported to associate with cardiovascular disease [33]. This unique group needs further clarification of its pathophysiology, and therapeutic targets since recent EMPA-KIDNEY outcomes also showed no benefit of adding sodium-glucose cotransporter 2 (SGLT2) inhibitors in this group [34].

As DKD is a risk factor for HF [6], we hypothesized that the presence of MAFLD would increase the risk of HF in this group [35]. The PU^−^DKD group showed a higher HHF risk when combined with MAFLD, although the difference was not statistically significant. Surprisingly, the PU^+^DKD group without MAFLD showed a higher HHF risk than those with MAFLD. Albuminuria has been associated with HF risk independent of eGFR [36]. Conversely, reduced eGFR was not significantly associated with incident heart failure at normal albuminuria levels [36]. Although MAFLD has potential mechanisms involved in HF risks, including low-grade inflammation [37], the direct effect of inflammation on the myocardium [38], and increased epicardial fat tissue [39, 40], proteinuria per se is a marker for generalized vascular endothelial dysfunction, which is likely to have a much stronger effect than that of fatty liver disease on HF [41]*.* Otherwise, insulin resistance might be a shared mechanism related to HF in patients with underlying DKD or MAFLD. Previously, Parente et al. reported that the waist-height ratio (WHR), a marker of central obesity [42], enhances the risk of HHF among patients with type 1 diabetes, regardless of proteinuria status [43] which is in contrast to our findings. There is a possibility that MAFLD did not affect patients with type 2 diabetes and DKD because they already had insulin resistance. However, it is expected that patients with type 1 diabetes have much less insulin resistance, which could explain the additive effect of WHR and DKD on the risk of HHF among patients with type 1 diabetes [42].

Of note, the effect of glucose-lowering drugs that modify the risk of HHF (e.g. SGLT2 inhibitors) were not calculated in this study. Because SGLT2 inhibitors are recommended to patients with DKD or HF due to its cardiorenal protective effects [44], DKD patients in our study might be exposed more to SGLT2 inhibitors than no-DKD patients that can lead to underestimate the risk of DKD on HHF.

Interventional strategies for high-risk populations should also be considered. The Asia–Pacific Working Party on Nonalcoholic Fatty Liver Disease or the American Association for the Study of Liver Diseases recommends that pioglitazone be considered in patients with nonalcoholic steatohepatitis [45]. However, our data suggested that individuals with MAFLD or DKD should be cautious about initiating pioglitazone due to the possible risk of heart failure. In this regard, glucose-lowering drugs, including SGLT2 inhibitors or glucagon-like peptide 1 receptor agonists (GLP-1 RAs) that confer protection against major cardiovascular diseases*,* are promising for preventing HF in NAFLD or MAFLD [46].

To the best of our knowledge, this is the first study to examine the effect of DKD and/or MAFLD on HHF in patients with diabetes with a relatively mild or moderate risk of CVD. To overcome the evaluation of eGFR and proteinuria status at a single point in time, we only included subjects with a stable DKD status over a two-year interval.

However, this study has some limitations. First, this study used claims data previously gathered for reimbursement purposes; thus, the diagnostic codes for certain patients might be incorrect. Second, another drawback is using a urinary dipstick test rather than a direct measure of urinary albumin excretion. The accuracy of the dipstick test may be affected by urine-specific gravity or pH [47]. In addition, it is not sensitive enough to detect microalbuminuria. Third, we could not adjust for several important variables (drugs affecting DKD or HF, laboratory tests for inflammation, and dietary habits except alcohol) and calculate indices such as AST to platelet ratio index or fibrosis-4 (FIB-4) index because of a lack of information. Fourth, we excluded the FLI score < 60 group as not having MAFLD, which might inadvertently categorize mild fatty liver disease as non-MAFLD. We also did not evaluate the advanced hepatic fibrosis or steatosis status. Recently, ADA guideline proposed algorithm using FIB-4 index for risk stratification in individuals with NAFLD or nonalcoholic steatohepatitis [48]. Further study should confirm the difference in fibrotic burden between MAFLD and its impact on HF outcomes. Finally, the clinical characteristics of patients with diabetes differ significantly across ethnic groups, and the results of this study cannot be directly applied to other ethnicities.

## Conclusion

Our study’s results indicated that DKD and/or MAFLD increased the risk of HHF. In line with ADA’s HF guidelines, we suggest that annual cardiac biomarker testing should be conducted at least in patients with DKD or MAFLD. In addition, interventional strategies, including treatment with SGLT2 inhibitors and GLP-1 RA, should be considered to prevent HF and ultimately reduce HF-related morbidity and mortality.

## Supplementary Information

**Additional file 1: Fig. S1.** Study population (A) and study design (B). **Table S1.** Incidence rate and risk of hospitalization for heart failure according to the DKD phenotype. **Table S2.** Incidence rate and risk of hospitalization for heart failure according to the DKD phenotype and MAFLD. **Table S3.** Incidence rate and risk of hospitalization for heart failure in the no-DKD group stratified by FLI.

## Data Availability

The data that support the findings of this study are available from the Korean National Health Insurance Service at https://nhiss.nhis.or.kr/bd/ab/bdaba021eng.do with the permission of the Korean National Health Insurance Service. Restrictions apply to the availability of these data, which were used under license for this study.

## Abbreviations

AST: Aspartate aminotransferase
ALT: Alanine aminotransferase
CKD: Chronic kidney disease
DKD: Diabetic kidney disease
eGFR: Estimated glomerular filtration rate
GGT: Gamma-glutamyl transferase
GLP-1 RA: Glucagon-like peptide 1 receptor agonist
HHF: Hospitalization for heart failure
ICD-10: International Classification of Disease-Tenth Revision
MAFLD: Metabolic dysfunction–associated fatty liver disease
SGLT2: Sodium-glucose cotransporter 2

## References

[1] Park JH, Ha KH, Kim BY, Lee JH, Kim DJ (2021). Trends in cardiovascular complications and mortality among patients with diabetes in South Korea. Diabetes Metab J.

[2] Jafari LA, Suen RM, Khan SS (2020). Refocusing on the primary prevention of heart failure. Curr Treat Options Cardiovasc Med.

[3] Savarese G, Lund LH (2017). Global public health burden of heart failure. Card Fail Rev.

[4] Levy D, Kenchaiah S, Larson MG, Benjamin EJ, Kupka MJ, Ho KK (2002). Long-term trends in the incidence of and survival with heart failure. N Engl J Med.

[5] Shah AD, Langenberg C, Rapsomaniki E, Denaxas S, Pujades-Rodriguez M, Gale CP (2015). Type 2 diabetes and incidence of cardiovascular diseases: a cohort study in 1.9 million people. Lancet Diabetes Endocrinol.

[6] Pop-Busui R, Januzzi JL, Bruemmer D, Butalia S, Green JB, Horton WB (2022). Heart failure: an underappreciated complication of diabetes. A consensus report of the American Diabetes Association. Diabetes Care.

[7] Dhingra R, Gaziano JM, Djousse L (2011). Chronic kidney disease and the risk of heart failure in men. Circ Heart Fail.

[8] Park J, Kim G, Kim H, Lee J, Lee YB, Jin SM (2021). The association of hepatic steatosis and fibrosis with heart failure and mortality. Cardiovasc Diabetol.

[9] Lee H, Kim G, Choi YJ, Huh BW, Lee BW, Kang ES (2020). Association between non-alcoholic steatohepatitis and left ventricular diastolic dysfunction in type 2 diabetes mellitus. Diabetes Metab J.

[10] Eslam M, Sanyal AJ, George J, International Consensus P (2020). MAFLD: a consensus-driven proposed nomenclature for metabolic associated fatty liver disease. Gastroenterology.

[11] Mendez-Sanchez N, Bugianesi E, Gish RG, Lammert F, Tilg H, Nguyen MH (2022). Global multi-stakeholder endorsement of the MAFLD definition. Lancet Gastroenterol Hepatol.

[12] Tsutsumi T, Eslam M, Kawaguchi T, Yamamura S, Kawaguchi A, Nakano D (2021). MAFLD better predicts the progression of atherosclerotic cardiovascular risk than NAFLD: Generalized estimating equation approach. Hepatol Res.

[13] Alharthi J, Gastaldelli A, Cua IH, Ghazinian H, Eslam M (2022). Metabolic dysfunction-associated fatty liver disease: a year in review. Curr Opin Gastroenterol.

[14] Berg DD, Wiviott SD, Scirica BM, Gurmu Y, Mosenzon O, Murphy SA (2019). Heart failure risk stratification and efficacy of sodium-glucose cotransporter-2 inhibitors in patients with type 2 diabetes mellitus. Circulation.

[15] National Health Insurance Data Sharing Service: Korean National Health Insurance Service; 2021. https://nhiss.nhis.or.kr/bd/ab/bdaba000eng.do.

[16] Kidney Disease: Improving Global Outcomes Diabetes Work Group (2020). KDIGO 2020 clinical practice guideline for diabetes management in chronic kidney disease. Kidney Int.

[17] Levey AS, Bosch JP, Lewis JB, Greene T, Rogers N, Roth D (1999). A more accurate method to estimate glomerular filtration rate from serum creatinine: a new prediction equation. Modification of Diet in Renal Disease Study Group. Ann Intern Med.

[18] Eslam M, Newsome PN, Sarin SK, Anstee QM, Targher G, Romero-Gomez M (2020). A new definition for metabolic dysfunction-associated fatty liver disease: an international expert consensus statement. J Hepatol.

[19] Bedogni G, Bellentani S, Miglioli L, Masutti F, Passalacqua M, Castiglione A (2006). The Fatty Liver Index: a simple and accurate predictor of hepatic steatosis in the general population. BMC Gastroenterol.

[20] Cho EJ, Jung GC, Kwak MS, Yang JI, Yim JY, Yu SJ (2021). Fatty liver index for predicting nonalcoholic fatty liver disease in an asymptomatic Korean population. Diagnostics.

[21] Choi HM, Park MS, Youn JC (2019). Update on heart failure management and future directions. Korean J Intern Med.

[22] Ponikowski P, Anker SD, AlHabib KF, Cowie MR, Force TL, Hu S (2014). Heart failure: preventing disease and death worldwide. ESC Heart Fail.

[23] Sun DQ, Jin Y, Wang TY, Zheng KI, Rios RS, Zhang HY (2021). MAFLD and risk of CKD. Metabolism.

[24] Lee H, Lee YH, Kim SU, Kim HC (2021). Metabolic dysfunction-associated fatty liver disease and incident cardiovascular disease risk: a nationwide cohort study. Clin Gastroenterol Hepatol.

[25] Chan KE, Koh TJL, Tang ASP, Quek J, Yong JN, Tay P (2022). Global prevalence and clinical characteristics of metabolic-associated fatty liver disease: a meta-analysis and systematic review of 10 739 607 individuals. J Clin Endocrinol Metab.

[26] Ahlqvist E, Storm P, Käräjämäki A, Martinell M, Dorkhan M, Carlsson A (2018). Novel subgroups of adult-onset diabetes and their association with outcomes: a data-driven cluster analysis of six variables. Lancet Diabetes Endocrinol.

[27] Targher G, Lonardo A, Byrne CD (2018). Nonalcoholic fatty liver disease and chronic vascular complications of diabetes mellitus. Nat Rev Endocrinol.

[28] Zhou YY, Zhou XD, Wu SJ, Hu XQ, Tang B, Poucke SV (2018). Synergistic increase in cardiovascular risk in diabetes mellitus with nonalcoholic fatty liver disease: a meta-analysis. Eur J Gastroenterol Hepatol.

[29] Salah HM, Pandey A, Van Spall HGC, Michos ED, McGarrah RW, Fudim M (2022). Meta-analysis of nonalcoholic fatty liver disease and incident heart failure. Am J Cardiol.

[30] Thomas MC, Brownlee M, Susztak K, Sharma K, Jandeleit-Dahm KA, Zoungas S (2015). Diabetic kidney disease. Nat Rev Dis Primers.

[31] Chang YT, Wu JL, Hsu CC, Wang JD, Sung JM (2014). Diabetes and end-stage renal disease synergistically contribute to increased incidence of cardiovascular events: a nationwide follow-up study during 1998–2009. Diabetes Care.

[32] Thomas MC, Macisaac RJ, Jerums G, Weekes A, Moran J, Shaw JE (2009). Nonalbuminuric renal impairment in type 2 diabetic patients and in the general population (national evaluation of the frequency of renal impairment cO-existing with NIDDM [NEFRON] 11). Diabetes Care.

[33] Buyadaa O, Magliano DJ, Salim A, Koye DN, Shaw JE (2020). Risk of rapid kidney function decline, all-cause mortality, and major cardiovascular events in nonalbuminuric chronic kidney disease in type 2 diabetes. Diabetes Care.

[34] Herrington WG, Staplin N, Wanner C, Green JB, Hauske SJ, The EMPA-KIDNEY Collaborative Group (2023). Empagliflozin in patients with chronic kidney disease. N Engl J Med.

[35] Mantovani A, Byrne CD, Benfari G, Bonapace S, Simon TG, Targher G (2022). Risk of heart failure in patients with nonalcoholic fatty liver disease: JACC review topic of the week. J Am Coll Cardiol.

[36] Blecker S, Matsushita K, Köttgen A, Loehr LR, Bertoni AG, Boulware LE (2011). High-normal albuminuria and risk of heart failure in the community. Am J Kidney Dis.

[37] Anstee QM, Mantovani A, Tilg H, Targher G (2018). Risk of cardiomyopathy and cardiac arrhythmias in patients with nonalcoholic fatty liver disease. Nat Rev Gastroenterol Hepatol.

[38] Luo B, Li B, Wang W, Liu X, Xia Y, Zhang C (2014). NLRP3 gene silencing ameliorates diabetic cardiomyopathy in a type 2 diabetes rat model. PLoS ONE.

[39] Emamat H, Tangestani H, Behrad Nasab M, Ghalandari H, Hekmatdoost A (2021). The association between epicardial adipose tissue and non-alcoholic fatty liver disease: a systematic review of existing human studies. EXCLI J.

[40] van Woerden G, Gorter TM, Westenbrink BD, Willems TP, van Veldhuisen DJ, Rienstra M (2018). Epicardial fat in heart failure patients with mid-range and preserved ejection fraction. Eur J Heart Fail.

[41] Deckert T, Feldt-Rasmussen B, Borch-Johnsen K, Jensen T, Kofoed-Enevoldsen A (1989). Albuminuria reflects widespread vascular damage. The Steno hypothesis. Diabetologia.

[42] Ashwell M, Hsieh SD (2005). Six reasons why the waist-to-height ratio is a rapid and effective global indicator for health risks of obesity and how its use could simplify the international public health message on obesity. Int J Food Sci Nutr.

[43] Parente EB, Harjutsalo V, Forsblom C, Groop PH, FinnDiane Study Group (2021). The impact of central obesity on the risk of hospitalization or death due to heart failure in type 1 diabetes: a 16-year cohort study. Cardiovasc Diabetol.

[44] Fontes-Carvalho R, Santos-Ferreira D, Raz I, Marx N, Ruschitzka F, Cosentino F (2022). Protective effects of SGLT-2 inhibitors across the cardiorenal continuum: two faces of the same coin. Eur J Prev Cardiol.

[45] Ando Y, Jou JH (2021). Nonalcoholic fatty liver disease and recent guideline updates. Clin Liver Dis.

[46] Yabiku K (2021). Efficacy of sodium-glucose cotransporter 2 inhibitors in patients with concurrent type 2 diabetes mellitus and non-alcoholic steatohepatitis: a review of the evidence. Front Endocrinol.

[47] White SL, Yu R, Craig JC, Polkinghorne KR, Atkins RC, Chadban SJ (2011). Diagnostic accuracy of urine dipsticks for detection of albuminuria in the general community. Am J Kidney Dis.

[48] ElSayed NA, Aleppo G, Aroda VR, Bannuru RR, Brown FM, Bruemmer D (2023). 4. Comprehensive medical evaluation and assessment of comorbidities: standards of care in diabetes-2023. Diabetes Care.

