# Supplementary material for: The effect of metabolic dysfunction-associated fatty liver disease and diabetic kidney disease on the risk of hospitalization of heart failure in type 2 diabetes: a retrospective cohort study
Source: Diabetol Metab Syndr. 2023 Mar 1;15:32. doi: 10.1186/s13098-023-01006-z (PMC9976518; doi:10.1186/s13098-023-01006-z)
Supplement: Supplementary file 1 — Additional file 1: Fig. S1. Study population (A) and study design (B). Table S1. Incidence rate and risk of hospitalization for heart failure according to the DKD phenotype. Table S2. Incidence rate and risk of hospitalization for heart failure according to the DKD phenotype and MAFLD. Table S3. Incidence rate and risk of hospitalization for heart failure in the no-DKD group stratified by FLI. [file 13098_2023_1006_MOESM1_ESM.docx]

Fig. S1. Study population (A) and study design (B).


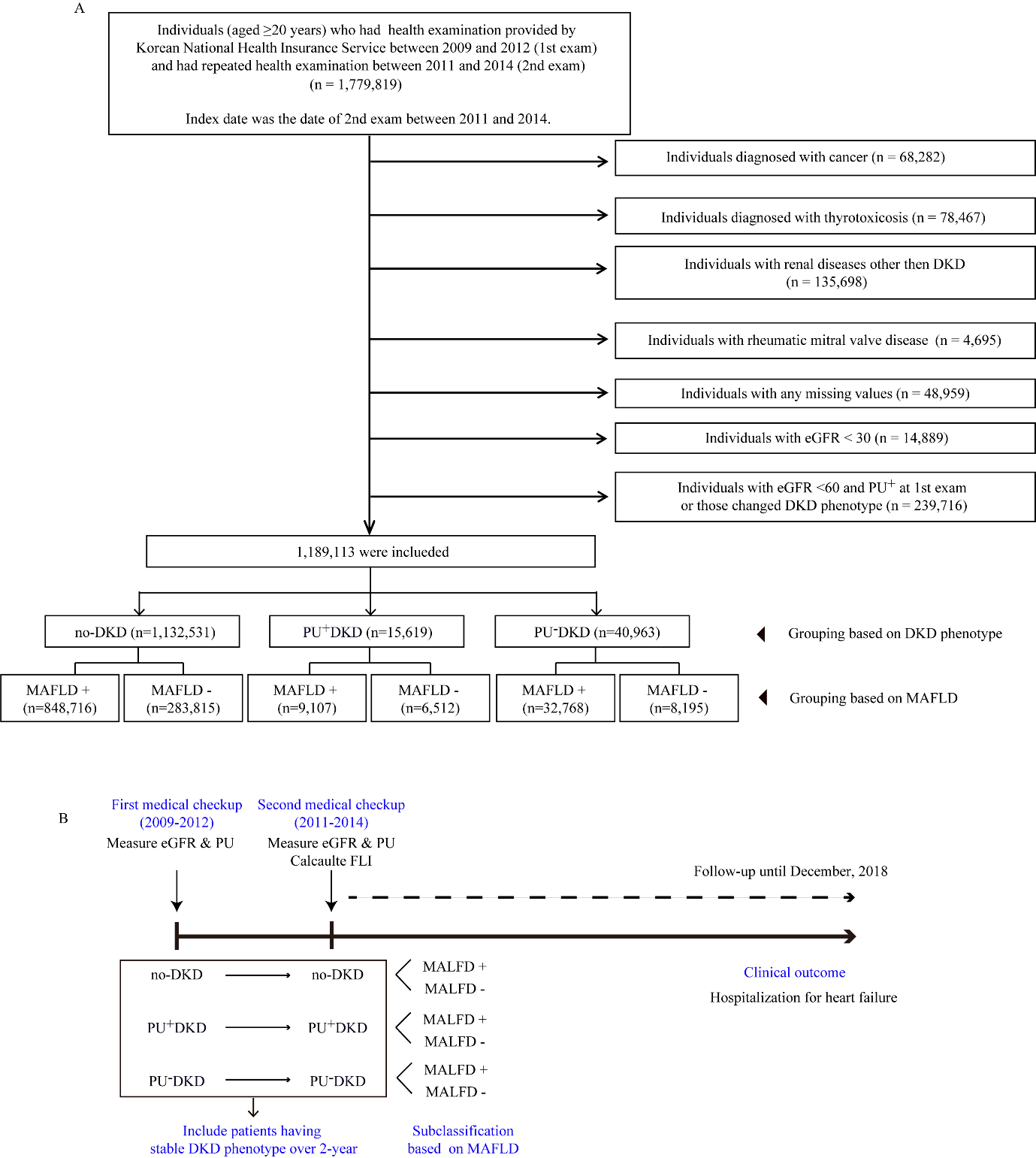


DKD, diabetic kidney disease; FLI, fatty liver index; MAFLD, metabolic dysfunction-associated fatty liver disease; PU, proteinuria.

no-DKD, normal eGFR (eGFR ≥ 60) with negative PU; PU^+^DKD, normal eGFR with positive PU; PU^-^DKD, reduced eGFR (eGFR <60) with negative PU.

Table S1. Incidence rate and risk of hospitalization for heart failure according to the DKD phenotype

|  | **no-DKD**  (n = 1,132,531) | **PU^+^DKD**  (n = 15,619) | **PU^-^DKD**  (n = 40,963) |
| --- | --- | --- | --- |
| HHF cases (n) | 4,479 | 257 | 1,045 |
| HHF incidence rate (per 1,000 person-year) | 0.60 | 2.64 | 4.14 |
| Model 1 HR (95% CI) | 1 (reference) | 4.45 (3.93,5.05) | 6.97 (6.51,7.45) |
| Model 2 HR (95% CI) | 1 (reference) | 4.25 (3.75,4.82) | 2.46 (2.30,2.64) |
| Model 3 HR (95% CI) | 1 (reference) | 4.18 (3.68,4.74) | 2.43 (2.26,2.61) |
| Model 4 HR (95% CI) | 1 (reference) | 3.59 (3.16,4.07) | 2.05 (1.91,2.20) |
| Model 5 HR (95% CI) | 1 (reference) | 3.12 (2.75,3.55) | 1.85 (1.73,1.99) |

no-DKD, normal eGFR (eGFR ≥ 60) with negative PU; PU^+^DKD, normal eGFR with positive PU; PU^-^DKD, reduced

eGFR (eGFR <60) with negative PU.

Model 1: Unadjusted;

Model 2: Adjusted for age and sex.

Model 3: Model 2 + smoking, alcohol consumption, and exercise

Model 4: Model 3 + hypertension, dyslipidemia, atrial fibrillation, and ischemic heart disease.

Model 5: Model 4 + fasting plasma glucose, diabetes duration, hemoglobin levels, and insulin use

Table S2. Incidence rate and risk of hospitalization for heart failure according to the DKD phenotype and MAFLD

|  | **no-DKD** | | **PU^+^DKD** | | **PU^-^DKD** | |
| --- | --- | --- | --- | --- | --- | --- |
|  | **MAFLD-**  (n = 848,716) | **MAFLD+**  (n = 283,815) | **MAFLD-**  (n = 9,107) | **MAFLD+**  (n = 6,512) | **MAFLD-**  (n = 32,768) | **MAFLD+**  (n = 8,195) |
| HHF cases (n) | 3,462 | 1,017 | 189 | 68 | 842 | 203 |
| HHF incidence rate  (per 1,000 person-year) | 0.62 | 0.54 | 3.35 | 1.65 | 4.18 | 3.99 |
| Model 1 HR (95% CI) | 1 (reference) | 0.88 (0.82,0.94) | 5.50 (4.75,6.36) | 2.71 (2.13,3.44) | 6.82 (6.32,7.35) | 6.50 (5.64,7.49) |
| Model 2 HR (95% CI) | 1 (reference) | 1.44 (1.34,1.55) | 4.75 (4.10,5.50) | 4.20 (3.30,5.34) | 2.51 (2.33,2.72) | 3.00 (2.61,3.46) |
| Model 3 HR (95% CI) | 1 (reference) | 1.45 (1.35,1.56) | 4.67 (4.03,5.41) | 4.16 (3.27,5.29) | 2.47 (2.29,2.67) | 2.97 (2.58,3.43) |
| Model 4 HR (95% CI) | 1 (reference) | 1.36 (1.26,1.46) | 4.05 (3.49,4.69) | 3.41 (2.68,4.35) | 2.11 (1.95,2.28) | 2.36 (2.05,2.73) |
| Model 5 HR (95% CI) | 1 (reference) | 1.41 (1.31,1.52) | 3.46 (2.98,4.01) | 3.18 (2.50,4.05) | 1.89 (1.75,2.05) | 2.19 (1.90,2.53) |

no-DKD, normal eGFR (eGFR ≥ 60) with negative PU; PU^+^DKD, normal eGFR with positive PU; PU^-^DKD, reduced eGFR (eGFR <60) with negative PU. MAFLD, metabolic dysfunction-associated fatty liver disease

Model 1: Unadjusted;

Model 2: Adjusted for age and sex.

Model 3: Model 2 + smoking, alcohol consumption, and exercise

Model 4: Model 3 + hypertension, dyslipidemia, atrial fibrillation, and ischemic heart disease.

Model 5: Model 4 + fasting plasma glucose, diabetes duration, hemoglobin levels, and insulin use

Table S3. Incidence rate and risk of hospitalization for heart failure in the no-DKD group stratified by FLI

|  | **no-DKD** | | |
| --- | --- | --- | --- |
|  | **FLI<30**  (n = 476,399) | **30≤FLI<60**  (n = 372,317) | **FLI≥60**  (n = 283,815) |
| HHF cases (n) | 1,964 | 1,498 | 1,017 |
| HHF incidence rate (per 1,000 person-year) | 0.63 | 0.61 | 0.54 |
| Model 1 HR (95% CI) | 1 (reference) | 0.97 (0.91,1.04) | 0.87 (0.81,0.94) |
| Model 2 HR (95% CI) | 1 (reference) | 1.12 (1.05,1.20) | 1.53 (1.42,1.66) |
| Model 3 HR (95% CI) | 1 (reference) | 1.13 (1.05,1.21) | 1.55 (1.43,1.67) |
| Model 4 HR (95% CI) | 1 (reference) | 1.06 (0.99,1.13) | 1.41 (1.30,1.52) |
| Model 5 HR (95% CI) | 1 (reference) | 1.10 (1.03,1.18) | 1.49 (1.37,1.62) |

FLI, fatty liver index.

Model 1: Unadjusted;

Model 2: Adjusted for age and sex.

Model 3: Model 2 + smoking, alcohol consumption, and exercise

Model 4: Model 3 + hypertension, dyslipidemia, atrial fibrillation, and ischemic heart disease.

Model 5: Model 4 + fasting plasma glucose, diabetes duration, hemoglobin levels, and insulin use
